# Estimated small dense low-density lipoprotein cholesterol and incident myocardial infarction in patients undergoing dialysis: a nationwide cohort study

**DOI:** 10.1080/07853890.2026.2745096

**Published:** 2026-10-09

**Authors:** Byung Sik Kim, Hyun-Jin Kim, Jiyeong Kim, Nayeon Choi, Jeong-Hun Shin

**Affiliations:** ^a^ Department of Internal Medicine, Hanyang University College of Medicine, Hanyang University Guri Hospital, Guri, Republic of Korea; ^b^ Biostatistics Lab, Medical Research Collaborating Center, Industry-University Cooperation Foundation, Hanyang University, Seoul, Republic of Korea; ^c^ Department of Pre-Medicine, College of Medicine, Hanyang University, Seoul, Republic of Korea

**Keywords:** Small dense low-density lipoprotein cholesterol, end-stage kidney disease, dialysis, myocardial infarction, ischemic stroke, cardiovascular risk

## Abstract

**Background:**

Small dense low-density lipoprotein cholesterol (sdLDL-C) is an atherogenic LDL subfraction that may reflect lipid-related coronary risk beyond conventional cholesterol levels. Estimated sdLDL-C (E-sdLDL-C) is calculated from a standard lipid panel, but its prognostic relevance for myocardial infarction in patients undergoing dialysis remains uncertain.

**Methods:**

This nationwide cohort study included 21,671 adults with end-stage kidney disease (ESKD) receiving long-term dialysis who underwent health screening in 2009–2017. E-sdLDL-C was calculated from fasting lipid profiles and categorised into quartiles. Incident myocardial infarction and ischemic stroke were the primary and secondary outcomes, respectively, assessed through December 2022.

**Results:**

During 166,428 person-years, 993 myocardial infarctions occurred, with 9,426 deaths before myocardial infarction. Fully adjusted hazard ratios for myocardial infarction in Quartiles 2–4 versus Quartile 1 were 1.24 (95% CI, 1.01–1.52), 1.61 (1.32–1.96), and 1.44 (1.15–1.80), respectively. The association remained significant after accounting for competing mortality. For ischemic stroke, 1,249 events and 9,254 competing deaths occurred during 165,727 person-years, with no significant association with E-sdLDL-C after full adjustment. Adding E-sdLDL-C increased the C-index for myocardial infarction from 0.716 to 0.726 (Δ = 0.010), without significant differences from models including LDL-C (0.725; *p* = 0.453) or non-HDL-C (0.727; *p* = 0.264).

**Conclusion:**

Among patients undergoing long-term dialysis, E-sdLDL-C was associated with incident myocardial infarction after multivariable adjustment, whereas no significant association was observed with ischemic stroke. These findings support the potential relevance of E-sdLDL-C for myocardial infarction risk assessment in patients with ESKD, although its clinical utility requires further validation.

## KEY MESSAGES

1. Higher E-sdLDL-C was associated with incident myocardial infarction in patients undergoing long-term dialysis, even after adjustment for LDL-C.
2. No significant association between E-sdLDL-C and ischemic stroke was observed after full adjustment.
3. E-sdLDL-C may provide complementary information for coronary risk stratification in end-stage kidney disease, but it should not replace established lipid measures.

## GRAPHICAL ABSTRACT

**Figure:**
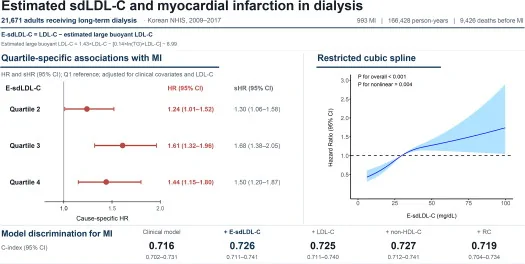

## Introduction

Ischemic heart disease is a major cardiovascular complication in patients with end-stage kidney disease (ESKD) who are undergoing dialysis. The prevalence of ischemic heart disease at the initiation of dialysis reportedly ranges from 15%–73%; myocardial infarction in the presence of uraemia is associated with poor short- and long-term prognosis [1,2]. Considering the central role of atherosclerosis in ischemic heart disease, low-density lipoprotein cholesterol (LDL-C) remains the principal lipid-related causal factor and therapeutic target for coronary risk reduction [3,4]. However, the role of conventional lipid markers and LDL-C-lowering therapy is less clear in patients already undergoing maintenance dialysis. Randomised trials of statin initiation in patients undergoing haemodialysis have shown limited reductions in the incidence of major cardiovascular events; moreover, current kidney disease guidelines do not recommend initiating statins in most patients on maintenance dialysis [4–7]. In addition, a “reverse epidemiology” of cholesterol has been observed in patients with ESKD, in whom lower cholesterol levels are associated with higher mortality, particularly in the setting of inflammation and malnutrition [2,8]. Accordingly, conventional lipid measures may not fully reflect lipid-related cardiovascular risk in this population [9].

Patients with ESKD undergoing dialysis often exhibit a distinct dyslipidaemic pattern characterised by hypertriglyceridemia, reduced high-density lipoprotein cholesterol (HDL-C) levels, qualitative alterations in LDL particles, and normal or even low LDL-C levels [10,11]. In patients with advanced kidney disease, impaired catabolism of triglyceride-rich lipoproteins, uremic milieu, inflammation, and oxidative stress promote the accumulation and modification of atherogenic lipoprotein particles [12,13]. These features provide a rationale for evaluating lipid markers that reflect lipoprotein particle composition or triglyceride-related remodelling beyond conventional cholesterol measures in patients undergoing dialysis.

Small dense LDL particles are considered particularly atherogenic because of their prolonged residence time, reduced LDL receptor affinity, greater arterial wall penetration, and higher susceptibility to oxidative modification [14,15]. Although direct measurement of small dense LDL cholesterol (sdLDL-C) is not routinely available, a recently proposed equation allows estimation of sdLDL-C from LDL-C and triglyceride concentrations routinely available from a standard lipid panel [16]. Recent large-scale cohorts have shown that the estimated sdLDL-C (E-sdLDL-C) level is independently associated with the incidence of cardiovascular disease and may improve risk stratification beyond that provided by conventional lipid indices [17,18]. In addition, evidence from chronic kidney disease cohorts suggests that a higher E-sdLDL-C level may be associated with adverse kidney and cardiovascular outcomes [19]. However, its association with incident cardiovascular events in patients receiving maintenance dialysis remains unclear.

We therefore conducted a nationwide cohort study using the Korean National Health Insurance Service (NHIS) database to evaluate the association between the baseline E-sdLDL-C level and the incidence of myocardial infarction in adults receiving long-term dialysis. We also examined its association with ischemic stroke and compared its predictive performance with that of the LDL-C, non-HDL-C, and remnant cholesterol levels.

## Methods

### Data sources

This retrospective cohort study used data from the Korean NHIS database, a nationwide administrative claims and health screening database that covers nearly the entire Korean population [20–22]. The database contains demographic information, inpatient and outpatient claims, diagnostic and procedural codes based on the International Classification of Diseases, 10th Revision (ICD-10), prescription records, and results from the National Health Screening Program. The health-screening database includes standardised questionnaires, anthropometric measurements, blood pressure, and laboratory test results. De-identified individual-level data were obtained under NHIS data access approval.

### Study population

We identified adults aged 20 years or older who were diagnosed with ESKD between 2009 and 2017. Long-term dialysis was defined based on claims for haemodialysis procedure codes (O7020, O7021, or O9991) or a peritoneal dialysis procedure code (O707) for at least 90 consecutive days [9,10]. Among 129,335 patients with ESKD, 44,828 underwent a general health checkup after the ESKD diagnosis was established. Patients were excluded if they had a history of myocardial infarction, ischemic stroke, or coronary revascularisation, kidney transplantation, triglyceride levels of 400 mg/dL or higher, missing covariates, or invalid calculated remnant cholesterol or E-sdLDL-C values (≤0 mg/dL). The final analytic cohort included 21,671 patients. The date of the first health screening examination after the diagnosis of ESKD was established was defined as the index date. The participants were classified into quartiles according to baseline E-sdLDL-C levels: Quartile 1 (<22.97 mg/dL; *n* = 5,417), Quartile 2 (22.97–29.56 mg/dL; *n* = 5,418), Quartile 3 (29.57–38.19 mg/dL; *n* = 5,418), and Quartile 4 (≥38.20 mg/dL; *n* = 5,418). The overall selection process is shown in Figure 1.

**Figure 1.**
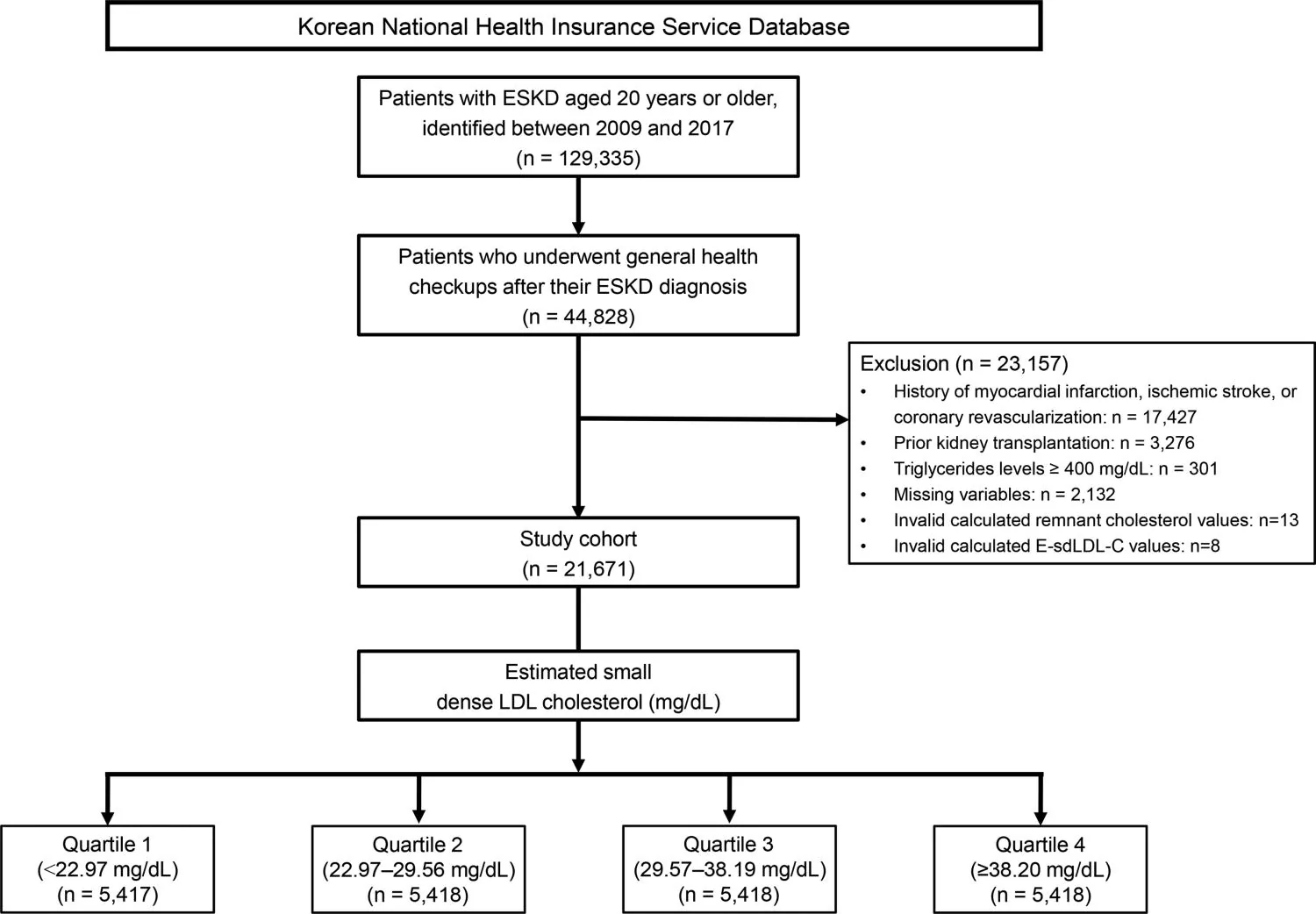
Flowchart of the study population selection. ESKD, end-stage kidney disease; LDL, low-density lipoprotein; sdLDL, small dense low-density lipoprotein.

### Calculation of lipid parameters

Fasting lipid profiles obtained at the index health screening examination were used to calculate lipid parameters. The LDL-C level was calculated using the Sampson equation [23]. Non-HDL-C was calculated as the total cholesterol minus the HDL-C, and remnant cholesterol was calculated as total cholesterol minus LDL-C minus HDL-C [9]. The E-sdLDL-C level was calculated using a standard lipid panel-based equation as follows: estimated large buoyant LDL-*C* = 1.43 × LDL-C − [0.14 × ln(triglyceride) × LDL-C] − 8.99; E-sdLDL-C = LDL-C − estimated large buoyant LDL-C [16]. Sampson LDL-C was used to calculate both remnant cholesterol and E-sdLDL-C in the primary analysis. This approach has been proposed as a practical method for estimating the sdLDL-C level from routinely available lipid measurements without requiring specialised assays for LDL subfraction analysis. A sensitivity analysis was performed using E-sdLDL-C calculated from LDL-C estimated by the Friedewald equation [24]. All the lipid values were expressed in mg/dL.

### Covariates

Baseline covariates were obtained from the index health-screening examination and claims data. The demographic variables measured included age, sex, and household income quartile. The lifestyle factors investigated included smoking status, alcohol consumption, and the frequency of physical activity. Body mass index was categorised as <18.5, 18.5 to 22.9, 23.0 to 24.9, or ≥25.0 kg/m^2^. The laboratory variables measured included fasting glucose level and lipid profile components. Hypertension and diabetes mellitus were defined using diagnostic codes and prescription records. The comorbidity burden was assessed using the Charlson Comorbidity Index [25]. Dialysis modality was classified as hemodialysis or peritoneal dialysis, and dialysis vintage was calculated as the interval between the first recorded ESKD date and the index health screening examination. Baseline medication use, including statins, triglyceride-lowering agents, antihypertensive agents, antidiabetic agents, and antiplatelet agents, was identified from prescription claims during the 1-year period before the index date. Statin use included both statin monotherapy and statin–ezetimibe combination therapy. The detailed definitions of the study population, comorbidities, medication classifications, and clinical outcomes are presented in Supplementary Table 1.

### Clinical outcomes

The primary outcome was incident myocardial infarction, and ischemic stroke was assessed as a secondary outcome. Myocardial infarction was defined as hospitalisation with ICD-10 codes I21 or I22 as the principal diagnosis accompanied by a coronary revascularisation procedure code. Ischemic stroke was defined as hospitalisation with ICD-10 codes I63 or I64 as the principal diagnosis accompanied by a brain imaging procedure code [9,26]. Participants were followed from the index date until outcome occurrence, death, or the end of the study period on December 31, 2022, whichever occurred first.

### Ethical approval

This study was reviewed by the Institutional Review Board of Hanyang University Guri Hospital and was determined to be exempt from full review (approval no. GURI 2026-04-041). The requirement for informed consent was waived because the study used de-identified retrospective data from the NHIS database. The study was conducted in accordance with the Declaration of Helsinki. Formal data analysis for the present study began on May 13, 2026, after confirmation of IRB exemption.

### Statistical analysis

Continuous variables are presented as means with standard deviations and categorical variables as numbers with percentages. Baseline characteristics were compared across the E-sdLDL-C quartiles using one-way analysis of variance or the chi-square test, as appropriate. Cumulative incidence functions were estimated with death before the target outcome treated as a competing event, and differences across E-sdLDL-C quartiles were assessed using Gray’s test. Kaplan–Meier curves were also generated and compared using the log-rank test. The incidence rates were calculated per 1,000 person-years of follow-up.

Cause-specific Cox proportional hazards models were used to estimate hazard ratios (HRs) and 95% confidence intervals (CIs), with Quartile 1 as the reference group. Model 1 was adjusted for age and sex. Model 2 was further adjusted for body mass index, smoking status, alcohol consumption, physical activity, household income, Charlson Comorbidity Index, hypertension, diabetes mellitus, statin use, triglyceride-lowering agent use, antiplatelet agent use, dialysis modality, and dialysis vintage. Model 3 was additionally adjusted for LDL-C. Fine–Gray subdistribution hazards models were also fitted using the same sequential adjustment models, with death before the target outcome treated as a competing event.

Restricted cubic spline analyses were performed to evaluate the continuous associations between lipid parameters and clinical outcomes after adjustment for clinical covariates included in Model 2. Spline analyses were performed using three knots for each lipid parameter and included E-sdLDL-C, LDL-C, non-HDL-C, and remnant cholesterol. Subgroup analyses were conducted according to age, sex, hypertension, diabetes mellitus, statin use, and LDL-C category; the potential effect modification was assessed using interaction terms. Model discrimination was evaluated using the C-index after adding each lipid parameter to the baseline clinical model. C-index values, 95% CIs, and pairwise comparisons between models were estimated using the compareC package based on linear predictors from Cox proportional hazards models. All statistical analyses were performed using SAS Enterprise Guide version 7.1 (SAS Institute, Cary, NC, USA) and R version 4.0.3 (R Foundation for Statistical Computing, Vienna, Austria). All p-values were two-sided, and *p* < 0.05 was considered statistically significant.

## Results

### Baseline characteristics

The baseline characteristics according to the E-sdLDL-C quartiles are summarised in Table 1 **and** Table 2. The final cohort included 21,671 patients undergoing long-term dialysis, with similar numbers of patients across quartiles. The mean E-sdLDL-C levels from Quartile 1 to Quartile 4 were 18.6 ± 3.2, 26.2 ± 1.9, 33.6 ± 2.5, and 48.0 ± 9.4 mg/dL, respectively. Patients in the higher E-sdLDL-C quartiles were slightly older and more frequently female. Systolic blood pressure was modestly lower across increasing quartiles, whereas diastolic blood pressure was similar among groups. The levels of total cholesterol, triglycerides, LDL-C, non-HDL-C, and remnant cholesterol increased with higher E-sdLDL-C quartiles, whereas that of HDL-C decreased. Fasting glucose was highest in Quartile 4. The proportion of patients with obesity increased across quartiles, and the incidence of diabetes mellitus was highest in Quartile 4. Hypertension and antiplatelet agent use were slightly less frequent in higher E-sdLDL-C quartiles. Statin use decreased across quartiles, whereas triglyceride-lowering agent use increased. The proportion of patients receiving peritoneal dialysis increased across E-sdLDL-C quartiles, while dialysis vintage was slightly shorter in the higher quartiles. Current smoking was less common in higher quartiles, and alcohol consumption differed modestly across groups.

**Table 1.** Baseline characteristics according to the estimated small dense LDL cholesterol quartiles.

Total patients (*N* = 21,671)		Estimated small dense LDL cholesterol level (mg/dL)				p-value
		Quartile 1 (<22.97)	Quartile 2 (22.97–29.56)	Quartile 3 (29.57–38.19)	Quartile 4 (≥38.20)	
		(*n* = 5,417)	(*n* = 5,418)	(*n* = 5,418)	(*n* = 5,418)	
Age, years		57.3 ± 11.8	57.7 ± 11.7	57.8 ± 11.5	57.8 ± 11.2	0.041
Sex, n (%)						<.001
	Male	3910 (72.2)	3407 (62.9)	3169 (58.5)	2743 (50.6)	
	Female	1507 (27.8)	2011 (37.1)	2249 (41.5)	2675 (49.4)	
Blood pressure, mmHg						
	SBP	136.9 ± 20.4	135.5 ± 19.7	134.8 ± 20.1	134.0 ± 20.6	<.001
	DBP	79.4 ± 12.4	79.4 ± 11.9	79.4 ± 12.0	79.3 ± 12.3	0.977
Smoking, n (%)						<.001
	Never	3151 (58.2)	3317 (61.2)	3382 (62.4)	3645 (67.3)	
	Past	1469 (27.1)	1401 (25.9)	1376 (25.4)	1163 (21.5)	
	Current	797 (14.7)	700 (12.9)	660 (12.2)	610 (11.3)	
Alcohol consumption, times/week						<.001
	0	4762 (87.9)	4858 (89.7)	4879 (90.1)	4914 (90.7)	
	1–2	502 (9.3)	468 (8.6)	423 (7.8)	397 (7.3)	
	≥3	153 (2.8)	92 (1.7)	116 (2.1)	107 (2)	
Exercise, times/week						0.003
	0	3328 (61.4)	3407 (62.9)	3397 (62.7)	3466 (64)	
	1–2	940 (17.4)	963 (17.8)	971 (17.9)	980 (18.1)	
	3–4	652 (12)	635 (11.7)	597 (11)	591 (10.9)	
	≥5	497 (9.2)	413 (7.6)	453 (8.4)	381 (7)	
Body mass index, kg/m^2^						<.001
	<18.5	401 (7.4)	329 (6.1)	267 (4.9)	186 (3.4)	
	18.5–22.9	2943 (54.3)	2680 (49.5)	2388 (44.1)	2066 (38.1)	
	23–24.9	1115 (20.6)	1181 (21.8)	1228 (22.7)	1329 (24.5)	
	≥25	958 (17.7)	1228 (22.7)	1535 (28.3)	1837 (33.9)	
Household income, quartiles						<.001
	First	1949 (36)	1788 (33)	1764 (32.6)	1786 (33)	
	Second	989 (18.3)	933 (17.2)	1004 (18.5)	963 (17.8)	
	Third	1148 (21.2)	1271 (23.5)	1207 (22.3)	1285 (23.7)	
	Fourth	1331 (24.6)	1426 (26.3)	1443 (26.6)	1384 (25.5)	
Charlson Comorbidity Index						<.001
	≤3	1346 (24.8)	1472 (27.2)	1552 (28.6)	1475 (27.2)	
	4	817 (15.1)	862 (15.9)	837 (15.4)	824 (15.2)	
	5	834 (15.4)	875 (16.1)	886 (16.4)	913 (16.9)	
	6	800 (14.8)	786 (14.5)	763 (14.1)	778 (14.4)	
	≥7	1620 (29.9)	1423 (26.3)	1380 (25.5)	1428 (26.4)	
Hypertension		4520 (83.4)	4491 (82.9)	4420 (81.6)	4423 (81.6)	0.022
Diabetes mellitus		2134 (39.4)	2040 (37.7)	2043 (37.7)	2245 (41.4)	<.001
Statin use		1894 (35)	1721 (31.8)	1368 (25.2)	1289 (23.8)	<.001
TG lowering agent use		139 (2.6)	208 (3.8)	275 (5.1)	366 (6.8)	<.001
Antiplatelet agent use		3151 (58.2)	3170 (58.5)	3083 (56.9)	3040 (56.1)	0.041
Dialysis modality, n (%)						<.001
	HD	4883 (90.1)	4615 (85.2)	4388 (81)	4057 (74.9)	
	PD	534 (9.9)	803 (14.8)	1030 (19)	1361 (25.1)	
Dialysis vintage, years		2.2 ± 1.9	2.0 ± 1.8	2.0 ± 1.8	1.8 ± 1.6	<.001

Values are mean ± standard deviation or n (%).

DBP, diastolic blood pressure; HD, hemodialysis; PD, peritoneal dialysis; SBP, systolic blood pressure; TG, triglyceride.

**Table 2.** Baseline laboratory characteristics according to the estimated small dense LDL cholesterol quartiles.

Total patients (*N* = 21,671)	Estimated small dense LDL cholesterol level (mg/dL)				p-value
	Quartile 1 (<22.97)	Quartile 2 (22.97–29.56)	Quartile 3 (29.57–38.19)	Quartile 4 (≥38.20)	
	(*n* = 5,417)	(*n* = 5,418)	(*n* = 5,418)	(*n* = 5,418)	
Fasting glucose level, mg/dL	108.6 ± 47.2	110.9 ± 48.9	111.6 ± 46.4	119.0 ± 53.0	<.001
Total cholesterol level, mg/dL	130.8 ± 24.8	152.9 ± 23.3	173.0 ± 22.8	212.9 ± 35.8	<.001
HDL cholesterol level, mg/dL	52.1 ± 16.9	49.2 ± 15.1	46.8 ± 14.2	44.9 ± 12.9	<.001
Triglyceride level, mg/dL	72.0 ± 32.4	103.7 ± 39.6	133.1 ± 50.3	183.5 ± 65.3	<.001
Estimated sdLDL cholesterol level, mg/dL	18.6 ± 3.2	26.2 ± 1.9	33.6 ± 2.5	48.0 ± 9.4	<.001
LDL cholesterol level, mg/dL	64.3 ± 19.6	85.0 ± 18.8	102.8 ± 19.9	135.4 ± 32.9	<.001
Non-HDL cholesterol level, mg/dL	78.7 ± 17.5	103.7 ± 14.4	126.1 ± 14.5	168.0 ± 32.3	<.001
Remnant cholesterol, mg/dL	14.4 ± 4.7	18.7 ± 6.0	23.3 ± 7.8	32.7 ± 11.5	<.001

Values are mean ± standard deviation or n (%).

HDL, high-density lipoprotein; LDL, low-density lipoprotein; sdLDL, small dense low-density lipoprotein; TG, triglyceride.

### E-sdLDL-C level and incident myocardial infarction

During 166,428 person-years of follow-up, 993 myocardial infarctions occurred, and 9,426 deaths occurred before myocardial infarction. The cumulative incidence of myocardial infarction differed across E-sdLDL-C quartiles after accounting for death as a competing event (Gray’s test *p* < 0.001) (Figure 2A). Kaplan–Meier curves for myocardial infarction and ischemic stroke are presented in Supplementary Figure 1. In the fully adjusted cause-specific Cox model, the HRs for Quartiles 2, 3, and 4 versus Quartile 1 were 1.24 (95% CI, 1.01–1.52), 1.61 (95% CI, 1.32–1.96), and 1.44 (95% CI, 1.15–1.80), respectively. The corresponding sHRs from the Fine–Gray model were 1.30 (95% CI, 1.06–1.58), 1.68 (95% CI, 1.38–2.05), and 1.50 (95% CI, 1.20–1.87), respectively (Table 3 and Supplementary Table 2). Restricted cubic spline analysis adjusted for clinical covariates showed a significant nonlinear association between the E-sdLDL-C level and myocardial infarction (p for overall association <0.001; p for nonlinearity = 0.004). In additional analyses of comparator lipid markers, LDL-C and non-HDL-C levels also showed positive associations with myocardial infarction, whereas remnant cholesterol showed an early increase followed by a plateau (Figure 3). In the sensitivity analysis using E-sdLDL-C calculated from Friedewald-estimated LDL-C, the association with myocardial infarction was consistent with the primary analysis (Supplementary Table 3).

**Figure 2.**
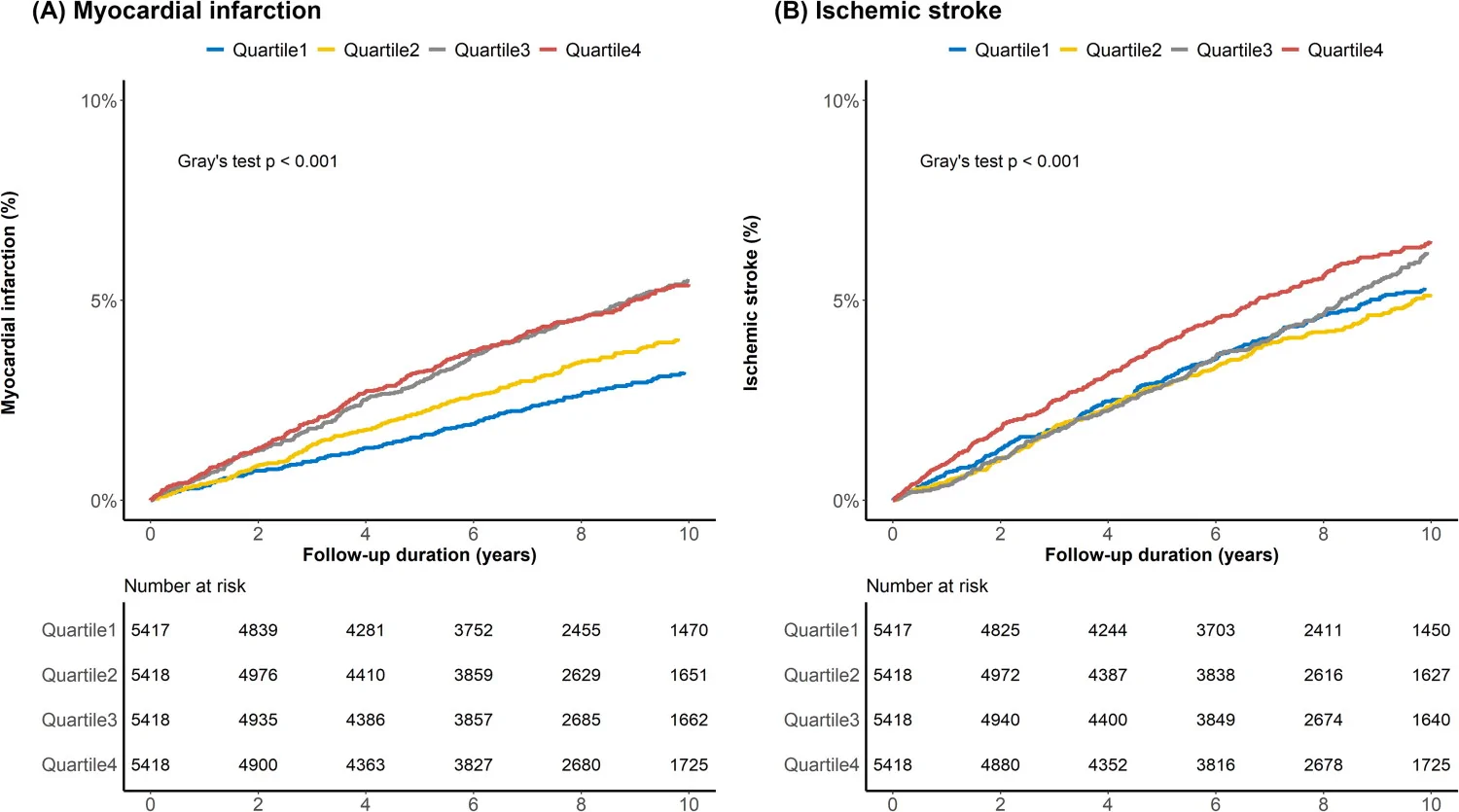
Cumulative incidence functions for myocardial infarction and ischemic stroke according to estimated small dense LDL cholesterol quartiles, accounting for death as a competing event. (A) Myocardial infarction. (B) Ischemic stroke. Differences in cumulative incidence functions across quartiles were assessed using Gray’s test.

**Figure 3.**
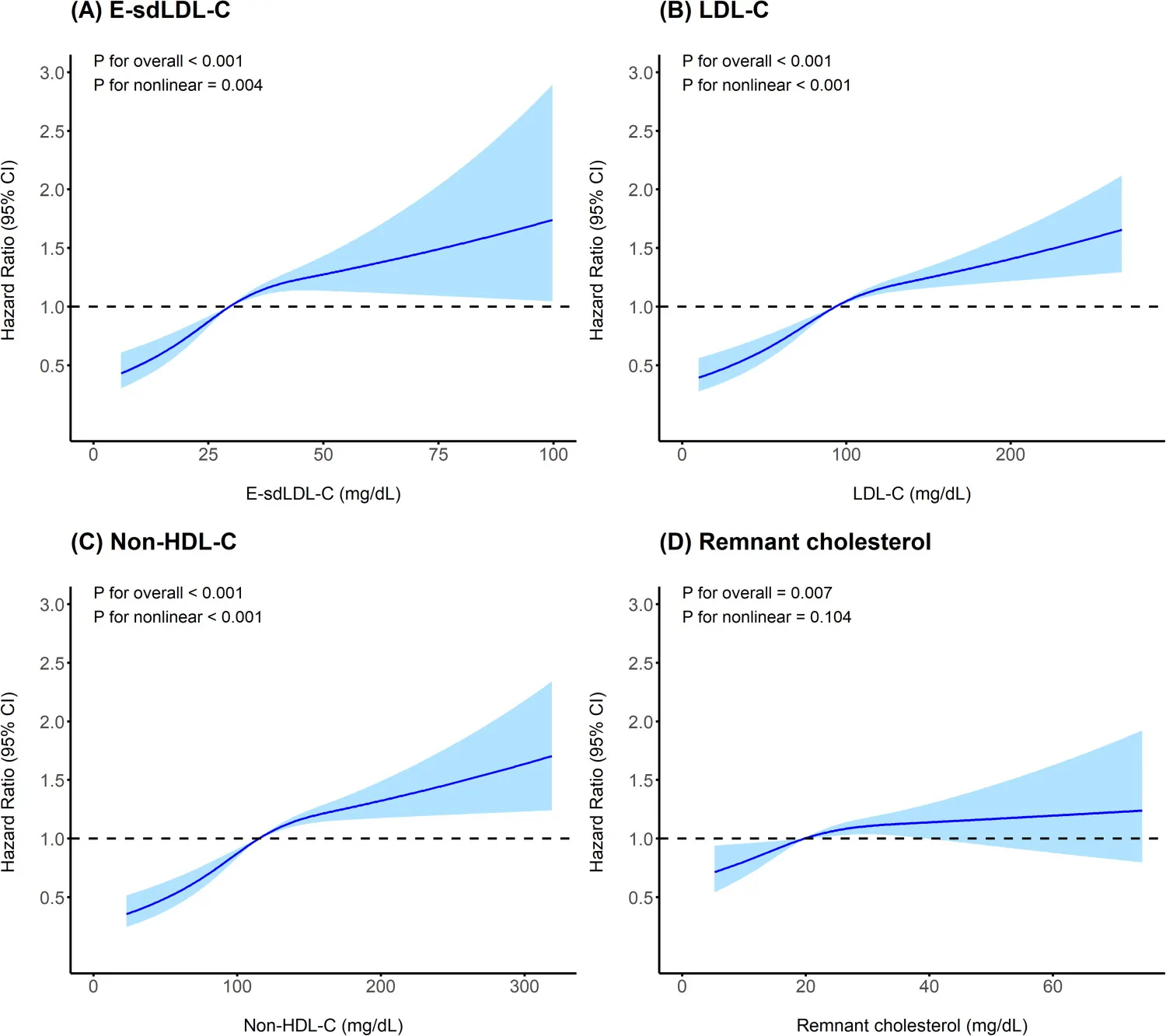
Restricted cubic spline analyses depicting the continuous associations between lipid markers and myocardial infarction. (A) estimated small dense LDL cholesterol (B) LDL cholesterol. (C) Non-HDL cholesterol. (D) Remnant cholesterol. Hazard ratios are shown with 95% confidence intervals. Models were adjusted for age, sex, body mass index, smoking status, alcohol consumption, exercise, household income, Charlson Comorbidity Index, hypertension, diabetes mellitus, statin use, triglyceride-lowering agent use, antiplatelet agent use, dialysis modality, and dialysis vintage.

**Table 3.** Risk of myocardial infarction and ischemic stroke according to the estimated small dense LDL cholesterol quartiles.

Total patients (*N* = 21,671)				Cause-specific Cox regression Model 1		Cause-specific Cox regression Model 2		Cause-specific Cox regression Model 3 (Model 2 + LDL-C)		Fine–Gray competing-risk regression Model 3	
E-sdLDL-C quartiles	Events	Person-years	IR	HR (95% CI)	p-value	HR (95% CI)	p-value	HR (95% CI)	p-value	sHR (95% CI)	p-value
Myocardial infarction											
Quartile 1 (*N* = 5,417)	174	40134	4.34	1 (Ref.)		1 (Ref.)		1 (Ref.)		1 (Ref.)	
Quartile 2 (*N* = 5,418)	226	41974	5.38	1.27 (1.04–1.55)	0.019	1.31 (1.07–1.60)	0.008	1.24 (1.01–1.52)	0.036	1.30 (1.06–1.58)	0.012
Quartile 3 (*N* = 5,418)	297	42132	7.05	1.68 (1.40–2.03)	<.001	1.78 (1.47–2.15)	<.001	1.61 (1.32–1.96)	<.001	1.68 (1.38–2.05)	<.001
Quartile 4 (*N* = 5,418)	296	42188	7.02	1.76 (1.45–2.12)	<.001	1.75 (1.44–2.13)	<.001	1.44 (1.15–1.80)	0.001	1.50 (1.20–1.87)	<.001
Ischemic stroke											
Quartile 1 (*N* = 5,417)	289	39806	7.26	1 (Ref.)		1 (Ref.)		1 (Ref.)		1 (Ref.)	
Quartile 2 (*N* = 5,418)	284	41775	6.80	0.92 (0.78–1.08)	0.287	0.93 (0.79–1.09)	0.357	0.89 (0.75–1.05)	0.157	0.94 (0.80–1.11)	0.475
Quartile 3 (*N* = 5,418)	329	42048	7.82	1.05 (0.90–1.23)	0.555	1.06 (0.90–1.25)	0.455	0.98 (0.82–1.17)	0.815	1.04 (0.88–1.24)	0.638
Quartile 4 (*N* = 5,418)	347	42098	8.24	1.13 (0.96–1.32)	0.145	1.09 (0.93–1.29)	0.289	0.94 (0.76–1.15)	0.516	0.99 (0.81–1.22)	0.955

CI, confidence interval; HR, hazard ratio; IR, incidence rate per 1,000 person-years; LDL-C, low-density lipoprotein cholesterol; Ref, reference; sHR, subdistribution hazard ratio.

Model 1: adjusted for age and sex.

Model 2: adjusted for age, sex, body mass index, smoking status, alcohol consumption, exercise, household income, Charlson Comorbidity Index, hypertension, diabetes mellitus, statin use, triglyceride lowering agent use, antiplatelet agent use, dialysis modality, and dialysis vintage.

Model 3: adjusted for all variables in Model 2 and LDL-C level.

Fine–Gray Model 3 used the same covariates as cause-specific Cox Model 3, with death treated as a competing event.

### E-sdLDL-C level and incidence of ischemic stroke

During 165,727 person-years of follow-up, 1,249 ischemic strokes occurred, and 9,254 deaths occurred before ischemic stroke. Although the cumulative incidence functions differed across E-sdLDL-C quartiles (Gray’s test *p* < 0.001) (Figure 2B), the association was no longer evident after multivariable adjustment. In the fully adjusted cause-specific Cox model, the HRs for Quartiles 2, 3, and 4 versus Quartile 1 were 0.89 (95% CI, 0.75–1.05), 0.98 (95% CI, 0.82–1.17), and 0.94 (95% CI, 0.76–1.15), respectively. The corresponding Fine–Gray estimates were also not significant (sHR, 0.94 [95% CI, 0.80–1.11], 1.04 [0.88–1.24], and 0.99 [0.81–1.22], respectively) (Table 3 and Supplementary Table 2). In spline analyses for ischemic stroke, E-sdLDL-C showed a modest overall association without evidence of nonlinearity, whereas LDL-C and non-HDL-C showed positive associations and remnant cholesterol showed no clear association (Supplementary Figure 2). Similarly, the sensitivity analysis using E-sdLDL-C calculated from Friedewald-estimated LDL-C showed no significant association with ischemic stroke after full adjustment (Supplementary Table 3).

### Subgroup and model discrimination analyses

Subgroup analyses for myocardial infarction showed no clear evidence of heterogeneity across the clinically relevant strata, including age, sex, hypertension, diabetes mellitus, statin use, and LDL-C category (Supplementary Figure 3). For ischemic stroke, the absence of a significant association was generally consistent across subgroups, with no consistent evidence of effect modification (Supplementary Figure 4).

In model discrimination analyses for myocardial infarction, adding E-sdLDL-C to the baseline clinical model increased the C-index from 0.716 (95% CI, 0.702–0.731) to 0.726 (95% CI, 0.711–0.741; *p* < 0.001 vs baseline). Discrimination was comparable to that of models including LDL-C (C-index, 0.725; *p* = 0.453 vs E-sdLDL-C) or non-HDL-C (C-index, 0.727; *p* = 0.264), and was greater than that of the model including remnant cholesterol (C-index, 0.719; *p* < 0.001) (Table 4). For ischemic stroke, adding E-sdLDL-C, LDL-C, or non-HDL-C resulted in only modest C-index improvement, whereas remnant cholesterol did not improve discrimination (Supplementary Table 4).

**Table 4.** Comparison of model discrimination for predicting myocardial infarction according to the inclusion of individual lipid markers.

Model	C-index (95% CI)	p-value vs (1)	p-value vs (2)	p-value vs (3)	p-value vs (4)
(1) Baseline	0.716 (0.702–0.731)				
(2) Baseline + estimated sdLDL cholesterol	0.726 (0.711–0.741)	<0.001			
(3) Baseline + LDL cholesterol	0.725 (0.711–0.740)	<0.001	0.453		
(4) Baseline + non-HDL cholesterol	0.727 (0.712–0.741)	<0.001	0.264	0.073	
(5) Baseline + remnant cholesterol	0.719 (0.704–0.734)	0.013	<0.001	0.002	<0.001

CI, confidence interval; HDL, high-density lipoprotein; LDL, low-density lipoprotein; sdLDL, small dense low-density lipoprotein.

The baseline model included age, sex, body mass index, smoking status, alcohol consumption, exercise, household income, Charlson Comorbidity Index, hypertension, diabetes mellitus, statin use, triglyceride-lowering agent use, antiplatelet agent use, dialysis modality, and dialysis vintage.

## Discussion

In this nationwide cohort study of 21,671 patients undergoing long-term dialysis, the E-sdLDL-C level was associated with the incidence of myocardial infarction. Compared with the lowest quartile, Quartiles 2–4 were associated with a higher risk of myocardial infarction after full multivariable adjustment, including LDL-C. The estimates were not monotonic across quartiles, with the highest point estimate observed in Quartile 3, consistent with the nonlinear association observed in the spline analysis. In contrast, no significant association was observed between the E-sdLDL-C level and ischemic stroke after full adjustment. These findings were consistent in the competing-risk analyses, with the association remaining evident for myocardial infarction but not for ischemic stroke after full adjustment. Addition of E-sdLDL-C modestly improved risk discrimination for myocardial infarction, with performance comparable to LDL-C and non-HDL-C and greater than remnant cholesterol. These findings suggest that the E-sdLDL-C level may be a practical marker of the coronary atherogenic risk in patients undergoing dialysis, but should not be interpreted as a superior replacement for established lipid measures.

The present findings extend the application of the recent evidence on E-sdLDL-C to a population in which lipid risk assessment remains clinically challenging. In a large UK Biobank primary prevention cohort, E-sdLDL-C level was found to be independently associated with the incidence of atherosclerotic cardiovascular disease, remained positively associated after adjustment for apolipoprotein B, and improved risk assessment beyond LDL-C and apolipoprotein B levels in discordance analyses [17]. International cohort analyses have also reported that E-sdLDL-C level predicts incident cardiovascular disease independent of conventional lipid parameters and may improve risk classification [18]. The evidence from populations with kidney disease is more limited, although a higher E-sdLDL-C level has been associated with ESKD and cardiovascular mortality in diabetic kidney disease [19]. However, cardiovascular risk in patients undergoing dialysis is influenced by a high burden of nontraditional factors, including the uremic milieu, inflammation, oxidative stress, volume overload, mineral bone disorder, and vascular calcification, all of which may complicate the interpretation of lipid markers in this population [2,27]. Our findings extend this evidence by demonstrating an association between E-sdLDL-C level and incident myocardial infarction in a large nationwide dialysis cohort. The present analysis used the same NHIS dialysis source population as our previous studies of LDL-C and remnant cholesterol, with substantial overlap in participants [9,10]. While those studies evaluated conventional LDL-C categories and remnant cholesterol, respectively, the present study focuses on E-sdLDL-C and compares its association and discriminative performance with LDL-C, non-HDL-C, and remnant cholesterol.

The stronger association with myocardial infarction than with ischemic stroke is clinically plausible. Myocardial infarction is more directly linked to coronary atherothrombosis, for which atherogenic LDL particle burden and LDL particle remodelling may have a central role [28]. In contrast, ischemic stroke in patients undergoing dialysis is mechanistically heterogeneous and may be driven by several factors, including atrial fibrillation, small-vessel disease, vascular calcification, blood pressure variability, dialysis-related hemodynamic stress, and competing risks in addition to atherosclerosis [29]. This outcome-specific pattern is consistent with prior observations suggesting that lipid-related risk may be more closely linked to coronary events than to ischemic stroke in patients with advanced kidney disease [7,30]. Therefore, the current data support a more specific role of E-sdLDL-C in coronary risk rather than a broad association with all vascular events.

Several mechanisms may explain the association between the E-sdLDL-C level and myocardial infarction. Small dense LDL particles have reduced LDL receptor affinity, longer plasma residence time, greater arterial wall entry and retention, and a higher susceptibility to oxidative modification; all these factors enhance their atherogenic potential [31]. In advanced kidney disease, delayed catabolism of triglyceride-rich lipoproteins and altered lipoprotein composition coexist with uraemia-related inflammation and oxidative stress, leading to accumulation and qualitative modification of atherogenic lipoprotein particles [8,12]. Since E-sdLDL-C is calculated from LDL-C and triglyceride-related terms, its close relationship with other atherogenic lipid measures is expected [16]. The association observed after full multivariable adjustment, including LDL-C, suggests that E-sdLDL-C may capture lipid-related risk not fully reflected by LDL-C alone, although this should not be interpreted as evidence that E-sdLDL-C represents a biologically independent lipid phenotype.

From a clinical perspective, E-sdLDL-C may provide a practical transformation of routinely available lipid measurements for myocardial infarction risk assessment in patients undergoing dialysis, without requiring additional laboratory testing. This may be particularly useful in large-scale screening databases and clinical settings where direct sdLDL-C measurement is not available. However, the improvement in discrimination was modest and comparable to that of LDL-C and non-HDL-C, and the clinical relevance of this small improvement remains uncertain. Therefore, E-sdLDL-C level should be interpreted as a complementary marker of lipid-related coronary risk rather than as a treatment target or a replacement for established lipid measures.

This study has several limitations that need to be acknowledged. First, the observational design precludes causal inference, and residual confounding from unmeasured factors such as dialysis adequacy, residual kidney function, inflammation, mineral bone disorder, or atrial fibrillation cannot be excluded. Second, eligibility required a health screening examination after the diagnosis of ESKD, which may have preferentially selected relatively healthier patients who were able to attend routine screening. This may limit the generalizability of our findings to the broader dialysis population. Third, the E-sdLDL-C level was calculated rather than directly measured, and the equation has not been specifically validated in patients undergoing dialysis. Fourth, the lipid parameters were based on the index health screening examination and may not reflect subsequent changes in lipid profiles or cumulative exposure during follow-up. In addition, lipid-lowering medication use was assessed only at baseline, and information on treatment intensity, adherence, or changes in therapy during follow-up was not available. Fifth, outcomes were defined using claims-based diagnostic and procedural codes, which may be subject to misclassification despite strict operational definitions. Sixth, although E-sdLDL-C level was found to be associated with myocardial infarction after LDL-C adjustment, it did not show superior discrimination compared to LDL-C or non-HDL-C. Finally, because this study included Korean adults, generalisability to other populations is limited and would require further validation and evidence.

In conclusion, higher E-sdLDL-C was associated with the incidence of myocardial infarction among patients undergoing long-term dialysis after multivariable adjustment, whereas no significant association was observed for ischemic stroke after full adjustment. E-sdLDL-C may serve as a complementary lipid-derived marker for myocardial infarction risk assessment in this population, although its clinical utility beyond established lipid measures requires further validation.

## Supplementary Material

supplementary.docx

## Data Availability

The data underlying this study are held by the NHIS. Access to these data is restricted and was granted to the authors under a data use agreement (Approval No. NHIS-2024-10-1-023). The datasets can be accessed through the NHIS (https://nhiss.nhis.or.kr) upon reasonable request and with approval from the NHIS.
