# Supplementary material for: Estimated small dense low-density lipoprotein cholesterol and incident myocardial infarction in patients undergoing dialysis: a nationwide cohort study
Source: Ann Med. 2026 Oct 9;58(1):2745096. doi: 10.1080/07853890.2026.2745096 (PMC13656698; doi:10.1080/07853890.2026.2745096)
Supplement: Supplementary file 1 [file IANN_A_2745096_SM9405.docx]

**Supplementary Materials**

**Supplementary Table 1.** Codes used to define the study population, comorbidities, medication history, and outcomes

**Supplementary Table 2.** Fine–Gray competing-risk analysis of myocardial infarction and ischemic stroke according to estimated small dense LDL cholesterol quartiles

**Supplementary Table 3.** Sensitivity analysis using estimated small dense LDL cholesterol calculated from LDL-C estimated by the Friedewald equation

**Supplementary Table 4.** Comparison of model discrimination for predicting ischemic stroke according to the inclusion of individual lipid markers

**Supplementary Figure 1.** Kaplan–Meier curves for myocardial infarction and ischemic stroke according to estimated small dense LDL cholesterol quartiles. (A) Myocardial infarction. (B) Ischemic stroke. Differences across quartiles were assessed using the log-rank test.

**Supplementary Figure 2.** Restricted cubic spline analyses depicting the continuous associations between lipid markers and ischemic stroke. (A) estimated small dense LDL cholesterol (B) LDL cholesterol. (C) Non-HDL cholesterol. (D) Remnant cholesterol.

**Supplementary Figure 3.** Subgroup analyses of the association between estimated small dense LDL cholesterol quartiles and the risk of myocardial infarction

**Supplementary Figure 4.** Subgroup analyses of the association between estimated small dense LDL cholesterol quartiles and the risk of ischemic stroke.

**Supplementary Table 1.** Codes used to define study populations, comorbidities, medication history, and outcomes

| Diagnosis | Definition |
| --- | --- |
| Haemodialysis | Defined as procedure codes O7020, O7021, or O9991 |
| Peritoneal dialysis | Defined as procedure code O707 |
| Myocardial infarction | Hospital admission with ICD-10 codes I21 or I22 as the primary diagnosis, accompanied by a coronary revascularisation procedure code |
| Coronary artery bypass graft surgery | Defined as procedure codes O1640–O1642, O1647–O1649, OA640–OA642, or OA647–OA649 |
| Percutaneous coronary intervention | Defined as procedure codes M6551–M6554, M6561–M6567, M6571, M6572, or O1876–O1877 |
| Coronary angiography | Defined as procedure code HA670 |
| Ischemic stroke | Hospital admission with ICD-10 codes I63 or I64 as the primary diagnosis, accompanied by a brain imaging procedure code |
| Brain imaging | Defined as procedure codes HA441, HA451, HA461, HA851, HE101, HE201, HE301, HE401, HE501, HE102, HE135, HE136, HE202, HE235, HE236, HE301, HE302, HE501, HE502, HE535, HE536, HI101, HI135, HI136, HI201, HI235, HI236, HI301, HI401, HI501, HI535, HI536, HJ101, HJ135, HJ136, HJ201, HJ235, HJ236, HJ301, HJ401, HJ501, HJ535, or HJ536 |
| Coronary revascularisation | Defined as the absence of diagnostic codes for myocardial infarction and the presence of a coronary revascularisation procedure code |
| Prior kidney transplantation | Defined as procedure code R3280 |
| Hypertension | Defined as ICD-10 codes I10–13 and I15 and prescription of antihypertensive agents |
| Diabetes mellitus | Defined as ICD-10 codes E10–14 and prescription of antidiabetic agents |
| **Cardiovascular death** | Defined as death due to cardiovascular disease (ICD-10 codes I00–I99) |
| **Medications** |  |
| Statin | Rosuvastatin, atorvastatin, simvastatin, pitavastatin, pravastatin, fluvastatin, lovastatin; statin–ezetimibe combination products were included in statin use |
| Triglyceride lowering agent | fenofibrate, bezafibrate, ciprofibrate, etofibrate, gemfibrozil, omega-3, nicotinic acid, acipimox |
| Antihypertensive agents | Amlodipine, nifedipine, nilvadipine, nimodipine, barnidipine, benidipine, cilnidipine, felodipine, isradipine, lacidipine, lercanidipine, manidipine, moxonidine, nicardipine, nitrendipine, nisoldipine, efonidipine, verapamil, diltiazem, azilsartan, candesartan, eprosartan, fimasartan, irbesartan, losartan, olmesartan, telmisartan, valsartan, alacepril, benazepril, captopril, cilazapril, enalapril, fosinopril, imidapril, lisinopril, moexipril, perindopril, ramipril, temocapril, zofenopril, carvedilol, atenolol, nebivolol, bisoprolol, metoprolol, amosulalol, arotinolol, betaxolol, bevantolol, celiprolol, labetalol, nadolol, propranolol, hydrochlorothiazide, chlorthalidone, tripamide, spironolactone, amiloride, furosemide, azosemide, torasemide, xipamide, metolazone, prazosin, cicletanine, clonidine, minoxidil, cadralazine |
| Antidiabetic agents | Metformin, gemigliptin, sitagliptin, vildagliptin, saxagliptin, linagliptin, teneligliptin, alogliptin, anagliptin, evogliptin, dapagliflozin, empagliflozin, ipragliflozin, ertugliflozin, enavogliflozin, acarbose, miglitol, voglibose, glibenclamide, gliclazide, glimepiride, glipizide, gliquidone, rosiglitazone, pioglitazone, lobeglitazone, repaglinide, nateglinide, mitiglinide, exenatide, lixisenatide, dulaglutide, albiglutide, insulin |
| Antiplatelet agent | Aspirin, clopidogrel, prasugrel, ticagrelor, cilostazol, dipyridamole, ticlopidine |
| Anticoagulants | Warfarin, rivaroxaban, dabigatran, apixaban, edoxaban |

Abbreviations: ICD-10, International Classification of Diseases-10^th^ revision.

**Supplementary Table 2.** Fine–Gray competing-risk analysis of myocardial infarction and ischemic stroke according to estimated small dense LDL cholesterol quartiles

| **Total patients (N=21,671)** | | | **Fine–Gray competing-risk regression Model 1** | | **Fine–Gray competing-risk regression Model 2** | | **Fine–Gray competing-risk regression Model 3 (Model 2 + LDL-C)** | |
| --- | --- | --- | --- | --- | --- | --- | --- | --- |
| **E-sdLDL-C quartiles** | **Events** | **Competing deaths** | **sHR (95% CI)** | **p-value** | **sHR (95% CI)** | **p-value** | **sHR (95% CI)** | **p-value** |
| **Myocardial infarction** | | | | | | | | |
| Quartile 1 (N=5,417) | 174 | 2,432 | 1 (Ref.) |  | 1 (Ref.) |  | 1 (Ref.) |  |
| Quartile 2 (N=5,418) | 226 | 2,285 | 1.33 (1.10–1.63) | 0.004 | 1.36 (1.11–1.66) | 0.003 | 1.30 (1.06–1.58) | 0.012 |
| Quartile 3 (N=5,418) | 297 | 2,342 | 1.79 (1.48–2.16) | <.001 | 1.85 (1.53–2.24) | <.001 | 1.68 (1.38–2.05) | <.001 |
| Quartile 4 (N=5,418) | 296 | 2,367 | 1.84 (1.53–2.22) | <.001 | 1.81 (1.49–2.20) | <.001 | 1.50 (1.20–1.87) | <.001 |
| **Ischemic stroke** | | | | | | | | |
| Quartile 1 (N=5,417) | 289 | 2,352 | 1 (Ref.) |  | 1 (Ref.) |  | 1 (Ref.) |  |
| Quartile 2 (N=5,418) | 284 | 2,248 | 0.96 (0.82–1.14) | 0.662 | 0.97 (0.82–1.14) | 0.682 | 0.94 (0.80–1.11) | 0.475 |
| Quartile 3 (N=5,418) | 329 | 2,331 | 1.11 (0.95–1.30) | 0.187 | 1.11 (0.95–1.31) | 0.201 | 1.04 (0.88–1.24) | 0.638 |
| Quartile 4 (N=5,418) | 347 | 2,323 | 1.19 (1.01–1.39) | 0.034 | 1.14 (0.97–1.35) | 0.113 | 0.99 (0.81–1.22) | 0.955 |

CI, confidence interval; LDL-C, low-density lipoprotein cholesterol; Ref, reference; sHR, subdistribution hazard ratio.

Death before the target outcome was treated as a competing event.

Model 1: adjusted for age and sex.

Model 2: adjusted for age, sex, body mass index, smoking status, alcohol consumption, exercise, household income, Charlson Comorbidity Index, hypertension, diabetes mellitus, statin use, triglyceride lowering agent use, antiplatelet agent use, dialysis modality, and dialysis vintage.

Model 3: adjusted for all variables in Model 2 and LDL-C level.

**Supplementary Table 3.** Sensitivity analysis using estimated small dense LDL cholesterol calculated from LDL-C estimated by the Friedewald equation

| **Total patients (N=21,671)** | | | **Cause-specific Cox regression Model 1** | | **Cause-specific Cox regression Model 2** | | **Cause-specific Cox regression Model 3 (Model 2 + LDL-C)** | |
| --- | --- | --- | --- | --- | --- | --- | --- | --- |
| **E-sdLDL-C** **quartiles** | **Events** | **Incidence rate** **(per 1,000 PY)** | **HR (95% CI)** | **p-value** | **HR (95% CI)** | **p-value** | **HR (95% CI)** | **p-value** |
| **Myocardial infarction** | | | | | | | | |
| Quartile 1 (N=5,418) | 180 | 4.49 | 1 (Ref.) |  | 1 (Ref.) |  | 1 (Ref.) |  |
| Quartile 2 (N=5,417) | 222 | 5.28 | 1.20 (0.98–1.46) | 0.072 | 1.24 (1.02–1.52) | 0.031 | 1.18 (0.97–1.44) | 0.103 |
| Quartile 3 (N=5,419) | 287 | 6.80 | 1.56 (1.30–1.89) | <.001 | 1.67 (1.38–2.02) | <.001 | 1.51 (1.24–1.85) | <.001 |
| Quartile 4 (N=5,417) | 304 | 7.21 | 1.74 (1.44–2.09) | <.001 | 1.77 (1.46–2.14) | <.001 | 1.47 (1.17–1.83) | <.001 |
| **Ischemic stroke** | | | | | | | | |
| Quartile 1 (N=5,418) | 287 | 7.22 | 1 (Ref.) |  | 1 (Ref.) |  | 1 (Ref.) |  |
| Quartile 2 (N=5,417) | 290 | 6.94 | 0.94 (0.80–1.11) | 0.470 | 0.96 (0.81–1.13) | 0.606 | 0.92 (0.78–1.09) | 0.319 |
| Quartile 3 (N=5,419) | 320 | 7.59 | 1.02 (0.87–1.20) | 0.771 | 1.05 (0.89–1.23) | 0.600 | 0.96 (0.81–1.15) | 0.684 |
| Quartile 4 (N=5,417) | 352 | 8.37 | 1.15 (0.98–1.34) | 0.094 | 1.13 (0.96–1.33) | 0.156 | 0.97 (0.79–1.19) | 0.759 |

CI, confidence interval; E-sdLDL-C, estimated small dense low-density lipoprotein cholesterol; HR, hazard ratio; LDL-C, low-density lipoprotein cholesterol; PY, person-years; Ref, reference.

LDL-C was estimated using the Friedewald equation and was used to calculate E-sdLDL-C in this sensitivity analysis.

Model 1: adjusted for age and sex.

Model 2: adjusted for age, sex, body mass index, smoking status, alcohol consumption, exercise, household income, Charlson Comorbidity Index, hypertension, diabetes mellitus, statin use, triglyceride-lowering agent use, antiplatelet agent use, dialysis modality, and dialysis vintage.

Model 3: Model 2 + LDL-C.

**Supplementary Table 4.** Comparison of model discrimination for predicting ischemic stroke according to the inclusion of individual lipid markers

| **Model** | **C-index (95% CI)** | **p-value vs (1)** | **p-value vs (2)** | **p-value vs (3)** | **p-value vs (4)** |
| --- | --- | --- | --- | --- | --- |
| (1) Baseline | 0.714 (0.701–0.727) |  |  |  |  |
| (2) Baseline + estimated sdLDL cholesterol | 0.716 (0.703–0.730) | 0.006 |  |  |  |
| (3) Baseline + LDL cholesterol | 0.716 (0.703–0.729) | 0.009 | 0.617 |  |  |
| (4) Baseline + non-HDL cholesterol | 0.715 (0.702–0.728) | 0.034 | 0.112 | 0.263 |  |
| (5) Baseline + remnant cholesterol | 0.714 (0.701–0.728) | 0.569 | 0.006 | 0.010 | 0.044 |

CI, confidence interval; HDL, high-density lipoprotein; LDL, low-density lipoprotein; sdLDL, small dense low-density lipoprotein.

The baseline model included age, sex, body mass index, smoking status, alcohol consumption, exercise, household income, Charlson Comorbidity Index, hypertension, diabetes mellitus, statin use, triglyceride-lowering agent use, antiplatelet agent use, dialysis modality, and dialysis vintage.

**Supplementary Figure 1.** Kaplan–Meier curves for myocardial infarction and ischemic stroke according to estimated small dense LDL cholesterol quartiles. (A) Myocardial infarction. (B) Ischemic stroke. Differences across quartiles were assessed using the log-rank test.

**Supplementary Figure 2.** Restricted cubic spline analyses depicting the continuous associations between lipid markers and ischemic stroke. (A) estimated small dense LDL cholesterol (B) LDL cholesterol. (C) Non-HDL cholesterol. (D) Remnant cholesterol. Hazard ratios are shown with 95% confidence intervals. Models were adjusted for age, sex, body mass index, smoking status, alcohol consumption, exercise, household income, Charlson Comorbidity Index, hypertension, diabetes mellitus, statin use, triglyceride-lowering agent use, dialysis modality, dialysis vintage, and antiplatelet agent use.

**Supplementary Figure 3.** Subgroup analyses of the association between estimated small dense LDL cholesterol quartiles and the risk of myocardial infarction

**Supplementary Figure 4.** Subgroup analyses of the association between estimated small dense LDL cholesterol quartiles and the risk of ischemic stroke
